# Creatinine, leucine, and tetrahydrocorticosterone emerge as potential biomarkers for the diagnosis of sarcopenic osteoarthritis in middle-aged and elderly individuals: a cross-sectional exploratory study

**DOI:** 10.3389/fmolb.2026.1743095

**Published:** 2026-02-11

**Authors:** Huihui Wu, Hui Gao, Liang Guo, Xinyu Feng, Zhishen Zhang, Yiding Zhao, Haihong Chen, Zhi Wang

**Affiliations:** 1 School of Gongli Hospital Medical Technology, University of Shanghai for Science and Technology, Shanghai, China; 2 Department of Orthopedics, Gongli Hospital of Shanghai Pudong New Area, Shanghai, China; 3 AigenX Biosciences Co., Ltd., Shanghai, China

**Keywords:** biomarkers, metabolomics, osteoarthritis, sarcopenia, serum

## Abstract

**Background:**

Osteoarthritis is a common degenerative joint disease that is often associated with age-related muscle wasting known as sarcopenia, particularly in the elderly. This comorbid condition, referred to as “sarcopenic osteoarthritis”, may have a distinct metabolic profile; however, specific serum biomarkers for this phenotype remain poorly characterized. Therefore, we conducted an untargeted serum metabolomics study to identify potential biomarkers of osteoarthritis in individuals with sarcopenia.

**Methods:**

This cross-sectional study enrolled 82 participants categorized into healthy controls, osteoarthritis, and sarcopenic osteoarthritis groups (n = 30, 30, 22). Fasting serum was analysed by untargeted LC-MS metabolomics. Differential metabolites were identified using multivariate statistics (PCA, PLS-DA; VIP >1) combined with univariate tests (P < 0.05, FDR-adjusted q < 0.05, |log_2_FC| ≥ 1). Enriched KEGG pathways were determined (P < 0.05).

**Results:**

Metabolomic analyses distinguished sarcopenic osteoarthritis from osteoarthritis alone by significant alterations in steroid hormone biosynthesis and sphingolipid metabolism. We screened a total of 20 substances for use as biomarkers of sarcopenic osteoarthritis and ended up focusing mainly on three of them.

**Conclusion:**

Untargeted serum metabolomics successfully distinguished between healthy controls, osteoarthritis, and sarcopenic osteoarthritis and identified several candidate biomarkers. Creatinine, leucine, and tetrahydrocorticosterone emerged as promising biomarkers for the detection and phenotyping of the distinct sarcopenic osteoarthritis phenotype based on their clinical relevance and ease of measurement.

## 1 Introduction

Osteoarthritis and sarcopenia are two closely associated age-related conditions whose coexistence significantly exacerbates adverse patient outcomes (Veronese et al., 2018). Global Burden of Disease (GBD) data indicate that osteoarthritis affects over 640 million individuals worldwide (GBD, 2021 Osteoarthritis Collaborators, 2023). Although sarcopenia is not separately categorised within the GBD framework, global consensus confirms its widespread prevalence among the elderly population (Cruz-Jentoft et al., 2019). Crucially, these conditions do not occur in isolation; epidemiological studies reveal that approximately 40% of patients with knee osteoarthritis also suffer from sarcopenia (Peng et al., 2024; Zhang et al., 2015; Dharmakulsakti et al., 2022). This comorbid state of “sarcopenic osteoarthritis” confers substantially greater clinical risks than either condition alone, such as a synergistic increase in fall risk by up to fourfold (Smith et al., 2024; Iijima and Aoyama, 2021). Consequently, elucidating the interactive and additive harm mechanisms when these diseases coexist is paramount for improving clinical management in high-risk populations.

The pathophysiological mechanisms of sarcopenic osteoarthritis involve complex interactions between the musculoskeletal system and metabolic disorders (Liao et al., 2023). This syndrome may arise from the superimposition of shared pathways between osteoarthritis and sarcopenia. Osteoarthritis is characterised by cartilage degeneration and low-grade inflammation (Wu et al., 2023), whilst sarcopenia is associated with age-related muscle protein breakdown, hormonal alterations, and chronic inflammation (Huang and Wang, 2021). Significant metabolic reorganisation is observable in both disease states, such as the shift of articular chondrocytes towards anaerobic glycolysis, alongside lipid metabolism abnormalities and impaired amino acid utilisation in muscle tissue (Zhai, 2019). Despite these overlapping mechanisms, objective biomarkers for diagnosing sarcopenic osteoarthritis remain elusive.

Metabolomics is a pivotal technology for comprehensive analysis of small-molecule metabolites within biological organisms, and serves as a potent instrument for identifying such biomarkers (Kell and Goodacre, 2014). While studies have separately revealed the metabolomic signatures of osteoarthritis or sarcopenia (Kameda et al., 2021; Hsu et al., 2024), systematic metabolomic research on the syndrome of sarcopenic osteoarthritis remains unexplored. We hypothesize that OS presents a distinct serum metabolomic fingerprint that differs from osteoarthritis alone. Therefore, this study aimed to: (a) compare serum metabolic profiles among healthy controls (H), osteoarthritis, and sarcopenic osteoarthritis groups using untargeted metabolomics; (b) identify differentially abundant metabolites and enriched pathways specific to the sarcopenic osteoarthritis phenotype; and (c) evaluate the potential of key metabolites as discriminative biomarkers for sarcopenic osteoarthritis.

## 2 Methods

### 2.1 Study design and population

This study was a cross-sectional observational investigation designed to compare serum metabolic profiles among elderly individuals with varying health statuses through metabolomic analysis. The research protocol received approval from the institutional ethics review board, with all participants providing signed informed consent. Subjects were patients aged ≥55 years diagnosed with symptomatic knee osteoarthritis (Kellgren-Lawrence grade ≥2) via knee radiography. All participants underwent grip strength testing and bioelectrical impedance analysis (BIA) to assess muscle status. Sarcopenia diagnosis strictly adhered to the European Working Group on Sarcopenia in the Elderly (EWGSOP2) guidelines: Initial screening for muscle strength was conducted using grip strength (men <27 kg, women <16 kg). Suspected cases underwent further BIA measurement of skeletal muscle mass in the limbs and calculation of the skeletal muscle mass index (SMI, kg/m^2^). BIA diagnosis employed the Asian standards recommended by EWGSOP2 (men <7.0 kg/m^2^, women <5.5 kg/m^2^).

**FIGURE 1:**
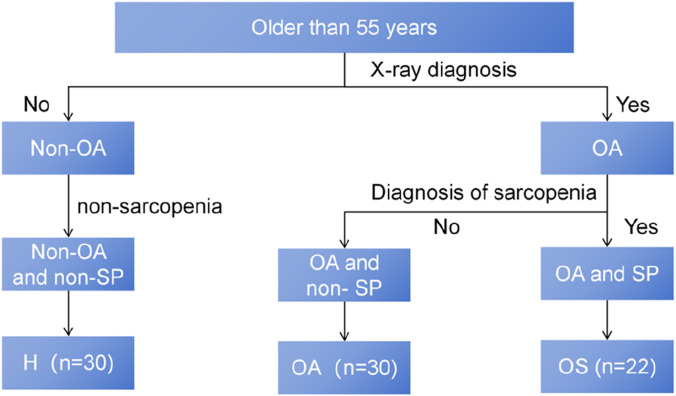
Subject Enrolment Flowchart. H, Healthy controls group; OA, Osteoarthritis group; OS, Sarcopenic osteoarthritis group.

Following this evaluation, subjects were stratified into three groups (Figure 1): sarcopenic osteoarthritis (OS) group (meeting diagnostic criteria for both sarcopenia and osteoarthritis), osteoarthritis (OA) group (meeting diagnostic criteria for osteoarthritis but not sarcopenia), and H group (age-matched healthy controls meeting neither set of criteria). It should be noted that the abbreviations H group, OA group, and OS group, as employed herein, refer solely to the study cohorts and should not be conflated with the broader diagnostic categories. The exclusion criteria encompassed: acute deep vein thrombosis or coagulation disorders; uncontrolled cardiovascular disease; recent surgery or trauma at the intended compression site on the subject’s limb; severe peripheral vascular disease or peripheral neuropathy; contraindications to exercise; conditions affecting exercise training implementation (e.g., stroke, craniofacial surgery history, major depression, or severe cognitive impairment); active cancer; known chronic kidney disease or an estimated glomerular filtration rate (eGFR) < 60 mL/min/1.73 m^2^. After applying this criterion, all included participants were confirmed to have eGFR ≥60 mL/min/1.73 m^2^ upon enrollment, and patients concurrently participating in other research projects. (Baseline characteristics of the 8 OS participants who declined blood sampling did not differ significantly from those of included OS participants; Supplementary Table S1).

The estimation of sample size was based on prior clinical trial literature that indicated 20 samples per group could detect significant metabolomic differences (Billoir et al., 2015). To control for individual variation and ensure statistical power, the target sample size per group was set at 30 cases. A total of 82 fasting venous serum samples were successfully collected and analysed (with 8 individuals in the OS group declining blood sampling). All samples were subjected to centrifugation and stored at −80 °C prior to non-targeted metabolomic analysis. By systematically comparing metabolite profiles across H, OA, and OS groups, this study aims to systematically reveal characteristic metabolic alterations and associated pathway disruptions in sarcopenic osteoarthritis, thereby providing a foundation for further exploration of its underlying pathological mechanisms.

### 2.2 Data collection and pretreatment of samples

We obtained data on gender, age, rheumatoid factor, anti-citrullinated protein antibody (ACPA), C-reactive protein, IL-6, and erythrocyte sedimentation rate from the patients’ medical records. Serum creatinine levels were measured, and eGFR was calculated for all participants using the CKD-EPI 2009 creatinine equation (Levey et al., 2009).

After patient enrollment, 2–5 mL of peripheral venous blood was collected, using additive-free blood collection tubes; mixed gently by inversion (avoiding vigorous shaking to prevent hemolysis); and allowed to stand at room temperature for approximately 1 h until coagulation and clot formation were observed. Subsequently, low-temperature, low-speed centrifugation (4,000 rpm, 10 min, 4 °C) was performed, and most of the supernatant (upper serum layer) was taken. If hemolysis occurred, the sample was unusable. 300 µL of serum was dispensed and stored at −80 °C for long-term storage until subsequent analytical processing.

The samples were thawed on ice and then placed in the Starlid™ automation workstation for metabolite extraction. A 100 µL aliquot of serum was transferred to an EP tube, followed by the addition of 400 µL of 80% methanol aqueous solution. The mixture was vortexed and incubated on ice for 5 min. Subsequently, the samples were centrifuged at 15,000 g for 20 min at 4 °C. An appropriate volume of the supernatant was collected and diluted with ultrapure water to achieve a final methanol concentration of 53%. The samples were then centrifuged again at 15,000 g for 20 min at 4 °C. The supernatant was collected and subjected to analysis by liquid chromatography-mass spectrometry (LC-MS). Metabolomics analysis was performed at AigenX Biosciences Co., Ltd. (Shanghai, China).

### 2.3 Data visualization and statistical analysis

Non-targeted metabolomics analysis was conducted to identify all metabolites in the serum samples from the H, OA, and OS groups. All analyses comparing metabolic differences between the OA and OS groups included body mass index (BMI) as a covariate in the model. Raw data were converted to mzXML format using Proteo Wizard, with metabolite identification performed via an in-house R package and the AigenXDB (V3.0) database. Quantitative analysis was completed using CD3.3 software. Statistical analysis employed MetaX software for data transformation, integrating multivariate and univariate methods. Principal Component Analysis (PCA) and Partial Least Squares Discriminant Analysis (PLS-DA) were first conducted to obtain Variable Importance Projection (VIP) values for metabolites. Concurrently, t-tests calculated inter-group significance and fold change (FC). Differential metabolites were screened using the criteria: VIP >1, P < 0.05, and |log_2_FC| ≥1 (i.e., FC ≥2 or FC ≤0.5). To account for multiple testing, the Benjamini–Hochberg false discovery rate (FDR) procedure was additionally applied; metabolites with q < 0.05 are reported in Supplementary Table S2. All downstream analyses, including the generation of Venn diagrams, K-means clustering, and biomarker evaluation, were performed using this FDR-significant metabolite set (q < 0.05). Functional annotation and pathway analysis of metabolites were performed using the KEGG database. Pathways were deemed significantly enriched when x/n > y/n and P < 0.05.

To evaluate the diagnostic efficacy of the candidate biomarkers, we performed receiver operating characteristic (ROC) curve analysis. First, ROC curves and corresponding areas under the curve (AUC) were calculated for each biomarker individually to distinguish between OA group and OS group. Concurrently, to assess the potential advantage of a combined diagnostic approach, we developed a multiple logistic regression model using the concentrations of the three metabolites as predictor variables and patient group (OA vs. OS) as the outcome. The model estimated the probability of each subject being classified as OS, and based on these probabilities, the ROC curve for the combined model was generated.Data processing throughout the analysis workflow primarily utilised Python (3.5.0/2.7.6), while statistical analysis and graphical presentation were accomplished using R (3.4.3) and tools such as gsea-3.0.

## 3 Results

### 3.1 Comparison of patient characteristics among the H, OA, and OS groups

Table 1 presents the baseline characteristics of the three groups. The sample size was 30 for H group, 30 for the OA group, and 22 for the OS group (α = 0.05). No significant differences in age or gender were observed between H and OA groups, or between the OA and OS groups (all p > 0.05).The levels of rheumatoid factor, erythrocyte sedimentation rate (ESR), and anti-cyclic citrullinated peptide (anti-CCP) antibody were negative in all participants, with no statistically significant differences. A statistically significant difference in BMI was observed between the OA group and OS group (p = 0.01). This difference is a recognized component of the sarcopenic phenotype and a potential confounder for systemic metabolism. The implications of this are addressed in the limitations section. Muscle mass reduction is a core feature of sarcopenia, and as muscle tissue is metabolically active, its loss can directly contribute to decreased body weight. Additionally, eGFR did not differ significantly between groups.

**TABLE 1:** Statistical tables of basic data.

characteristic	H (n = 30)	OA (n = 30)	OS (n = 22)	H vs. OA (p-value)	OA vs. OS (p-value)
Age (mean ± SD)	64.30 ± 8.47	67.00 ± 6.23	69.18 ± 9.49	0.13	0.19
Gender (mean ± SD)	0.40	0.40	0.36	1.00	0.79
BMI (mean ± SD)	24.83 ± 2.80	24.97 ± 2.77	22.66 ± 3.44	0.76	0.01
eGFR (mean ± SD, mL/min/1.73 m^2^)	88.7 ± 13.9	88.1 ± 14.5	85.6 ± 15.1	0.87	0.52
Rheumatoid factor present, n (%)	0	0	0	1	1
ESR, n (%)	0	0	0	1	1
Anti-CCP positive, n (%)	0	0	0	1	1

H, healthy controls group; OA, osteoarthritis group; OS, sarcopenic osteoarthritis group; H vs. OA, Group H versus OA; OA, vs. OS, Group OA, versus OS; BMI, body mass index; ESR, erythrocyte sedimentation rate; Anti-CCP, Anti-cyclic citrullinated peptide antibody; eGFR, estimated glomerular filtration rate.

### 3.2 Key biomarkers were identified by analyzing metabolic profiles

#### 3.2.1 The orthogonal partial least squares-discriminant analysis (OPLS-DA) was employed to assess the repeatability of the dataset

**FIGURE 2:**
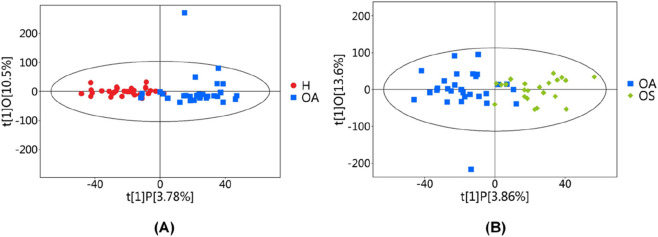
OPLS-DA score chart. H, Healthy controls group; OA, Osteoarthritis group; OS, Sarcopenic osteoarthritis group. Each point represents a single sample, and **(A)** Shows the OPLS-DA score plots for t H vs. OA, and **(B)** Shows the OPLS-DA score plots for OA vs. OS.

The OPLS-DA model (Figure 2) demonstrated distinct separation between groups and tight intra-group clustering, reflecting high reproducibility and significant inter-group divergence, which underscores the reliability of the findings.

#### 3.2.2 Screening for potential biomarkers of OS from differential metabolites

Table 2 shows that under the differential metabolite screening condition of VIP >1 and P-Value <0.05, 131 differential metabolites were detected in the H vs. OA, with 71 differential metabolites upregulated in expression, and up to 60 downregulated differential metabolites. Sixty differential metabolites were detected in the OA vs. OS, with 8 upregulated differential metabolites and 52 downregulated differential metabolites. We observed that the vast majority of metabolites in OA vs. OS exhibited a declining trend.

**TABLE 2:** Comprehensive statistical table of quantities of different differential metabolites.

Group	Cpd_all	Cpd_diff	Cpd_diff_up	Cpd_diff_down
H Vs. OA	1,474	131	71	60
OA vs. OS	1,474	60	8	52

Group, name of the comparison group; Cpd_all, total number of substances detected; Cpd_diff, number of different substances in the group; Cpd_diff_up, the number of different substances adjusted upward in the group; Cpd_diff_down, the number of different substances adjusted downward in the group; H, healthy controls group; OA, osteoarthritis group; OS, sarcopenic osteoarthritis group; H vs. OA, Group H versus OA; OA, vs. OS, Group OA, versus OS., Note: Differential metabolites were identified using VIP >1, P < 0.05, and |log_2_FC| ≥1. After applying FDR, adjustment (q < 0.05), 87 metabolites in H vs. OA, and 34 metabolites in OA, vs. OS, remained significant (Supplementary Table S2).

**FIGURE 3:**
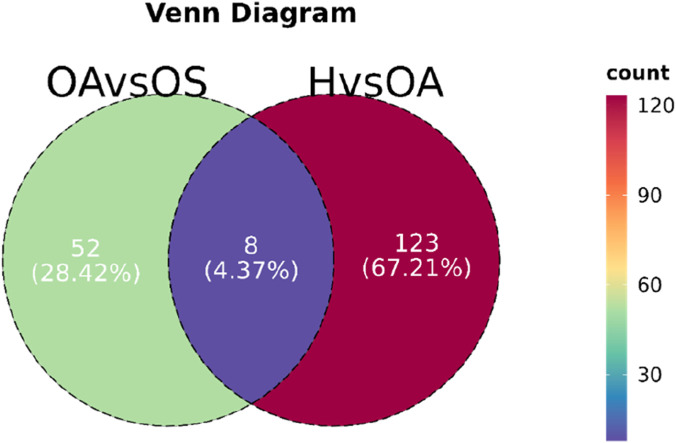
Venn diagrams of differential metabolites between groups. H: Healthy controls group; OA, Osteoarthritis group; OS, Sarcopenic osteoarthritis group; H vs. OA, Group H versus OA; OA vs. OS, Group OA versus OS.

Then we made Venn diagrams of the differential metabolites between two and two (Figure 3), there are 8 same differential metabolites between H vs. OA and OA vs. OS, with Creatinine, 4-Amino-2-oxo-1,2-dihydropyrimidine -5-carboxylic acid, 5,6-Dihydroxyindole-2-carboxylic acid, Methylimidazole acetaldehyde, 2-[4-Acetamido-3-[(4-chlorophenyl)thio]-2- methylindol-1-yl]acetic acid, Ethanolamine Phosphate, a total of 6 metabolic differences all increased and then decreased, and Prolyl-Histidine, 7H -Benzo [de]anthracen-7-one, a total of 2 differential metabolites decreased and then increased. We found that there were no consecutive differential metabolites with the same trend in these 8 metabolites, which can hardly serve as a basis for the progression from H group to OS group as a potential biomarker. Of these eight metabolites, none showed consecutive differential trends. While they could not be used as potential biomarkers for early-onset OS, subsequent studies could focus on these metabolites’ trends.

**FIGURE 4:**
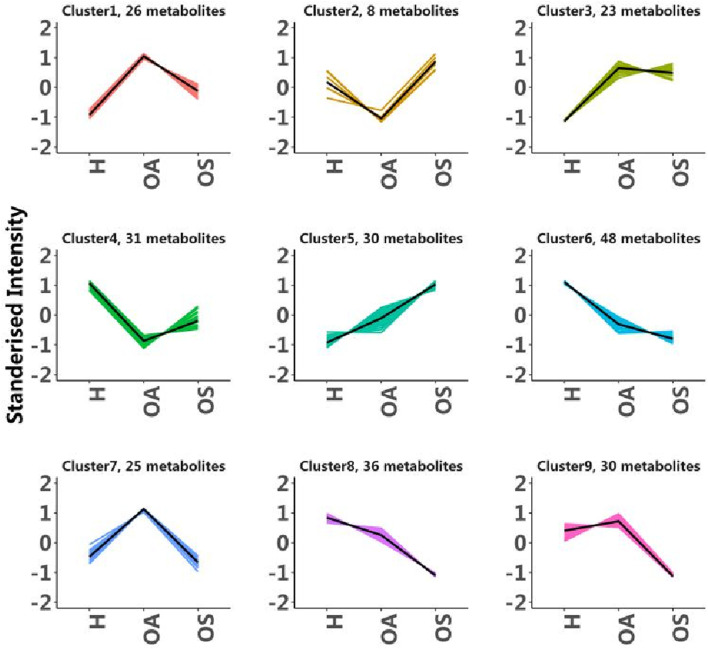
The K-means plot shows all groups. H, Healthy controls group; OA, Osteoarthritis group; OS, Sarcopenic osteoarthritis group. A cluster represents metabolites with the same trend, and the number of metabolites in a cluster is represented by *metabolite(s).

To investigate trends in metabolite content across different subgroups, the relative content of all comparisons was determined using screening criteria for all differential metabolites obtained by Z-score normalisation, followed by K-means cluster analysis (Figure 4). Firstly, we focused on metabolites with consistent trends of change from H group to the OA group and finally to the OS group, as these could be potential markers for diagnosing OS. These metabolites were found in clusters 5, 6, and 8 in Figure 4. Secondly, we focused on metabolites that did not change significantly from the H group to the OA group, but which showed significant differences in the OS group. In addition, 20 metabolites that can be used as potential biomarkers for OS were identified by screening the top 30 differential metabolites ranked by OA vs. OS.

**FIGURE 5:**
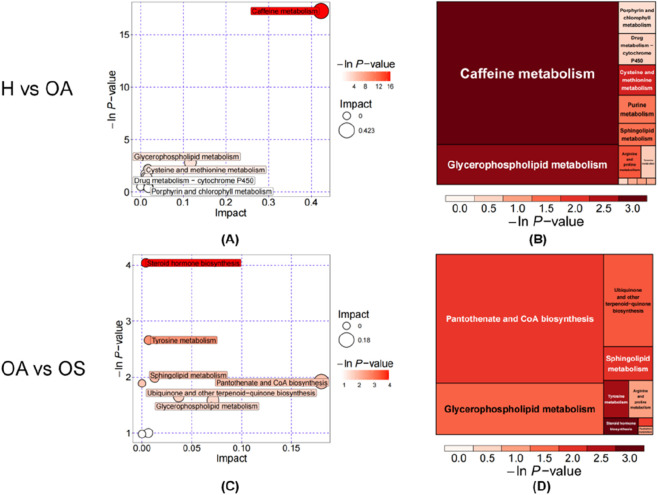
Metabolic pathway analysis of differential metabolites. H, Healthy controls group; OA, Osteoarthritis group; OS: Sarcopenic osteoarthritis group; H vs. OA, Group H versus OA; OA vs. OS: Group OA versus OS. **(A)** Differential pathway enrichment plot: H vs. OA. **(B)** Rectangular treemap of enriched pathways: H vs. OA. **(C)** Differential pathway enrichment plot: OA vs. OS. **(D)** Rectangular treemap of enriched pathways: OA vs. OS.

**FIGURE 6:**
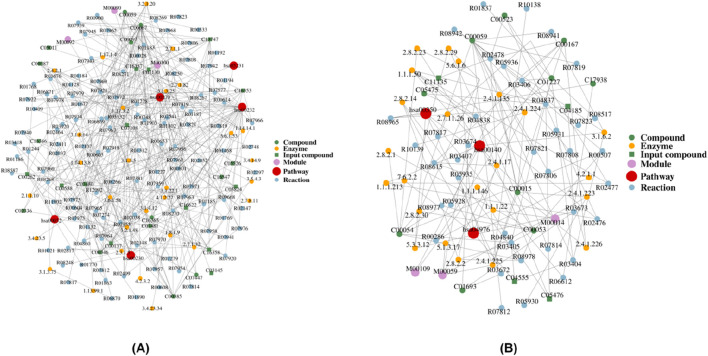
Regulatory network analysis of differential metabolites. **(A)** Differential metabolite regulatory network: H vs. OA. **(B)** Differential metabolite regulatory network: OA vs. OS.

We subsequently performed GO and KEGG analyses of all differential metabolites. The results are shown in Figure 5 and, when combined with network-based enrichment analysis (Figure 6), demonstrate that the H vs. OA is dominated by energy and base metabolism (e.g., caffeine, purine, and choline metabolism). The core region contains multiple metabolite and enzyme nodes, and these pathway nodes are concentrated in the central network region and connected to multiple metabolic reactions. This suggests that they are significantly affected in the H vs. OA. By contrast, the OA vs. OS shows a smaller, less complex network primarily influenced by hormone and bile metabolism (e.g., tyrosine metabolism, steroid hormones, and bile acids).

Among the nine potential biomarkers ultimately identified, we further focused on creatinine, leucine, and tetrahydrocorticosterone (THB). The selection criteria were threefold: first, these molecules reside within clusters demonstrating progressive changes associated with disease advancement (e.g., leucine in the consistently declining Cluster 8, THB in the Cluster 9); second, they possess well-defined biological relevance, directly reflecting muscle mass (creatinine), protein metabolism (leucine) and inflammation levels (THB); and third, they are either routinely measured or readily standardized in clinical laboratories, endowing them with high potential for clinical translation. All three survived FDR adjustment (q <0.05) in the OA vs. OS (Supplementary Table S2).

The three initially screened biomarkers (creatinine, leucine, and THB) exhibited significant differences between the OA and OS groups after FDR correction. However, considering the significant difference in BMI between the two groups and the potential confounding influence of renal function on creatinine levels, we performed additional sensitivity analyses to assess the impact of these confounders. When BMI was included as a covariate in the analysis of covariance model, the intergroup differences for all three metabolites remained statistically significant (all adjusted p-values <0.05; Supplementary Table S3). These findings indicate that the observed metabolic alterations are independently associated with sarcopenia and are more likely to reflect direct involvement in the underlying pathophysiological mechanisms of the condition.

**FIGURE 7:**
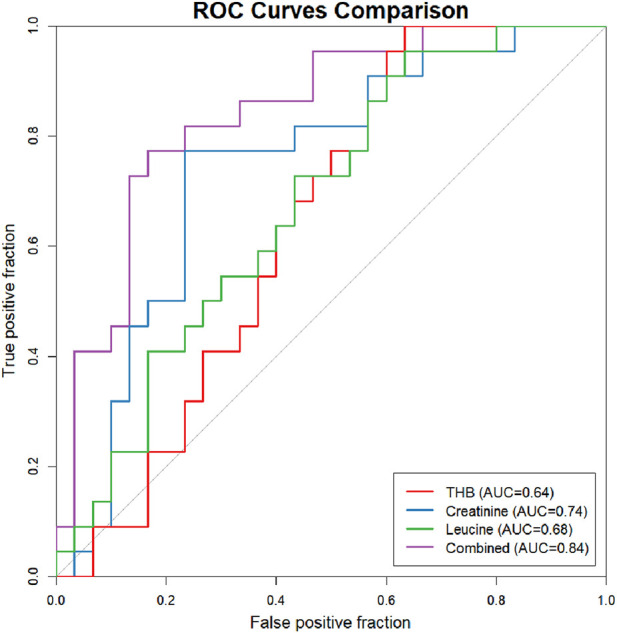
Receiver operating characteristic curves for creatinine, leucine, tetrahydrocorticosterone (THB) and the combination of three metabolites, along with their area under the curve (AUC) values for distinguishing OA from OS.

We further evaluated the diagnostic performance of the three candidate metabolites and their combined model in differentiating between the OA and OS groups (Figure 7). Among individual metabolites, creatinine showed the highest diagnostic accuracy with an AUC of 0.74, while leucine and THB yielded AUC values of 0.68 and 0.64, respectively. Notably, the combined logistic regression model incorporating creatinine, leucine, and THB demonstrated superior diagnostic performance, achieving an AUC of 0.84. From a predictive performance standpoint, these results provide further support for the selection of these three biomarkers based on their biological relevance and clinical utility.

## 4 Discussion

A substantial remodeling of energy metabolism was observed in the H vs. OA (Wu et al., 2023). The levels of several metabolites were significantly higher in the OA group. These results suggest that increased caffeine metabolic activity in osteoarthritis is due to the presence of these substances, which are caffeine or related xanthine metabolites, which is consistent with the abnormal caffeine metabolism in osteoarthritis found in genetic studies (Gu et al., 2023). Furthermore, alterations in the caffeine pathway may serve as a reflection of modulation of oxidative stress or cellular signaling, which could hold potential pathological significance in osteoarthritis (Li et al., 2025). Concurrently, nicotinamide and its derivatives exhibited a marked decrease in the OA group, indicating a potential disruption in NAD + metabolism. In addition, fluctuations in the levels of several amino acid dipeptides were observed, including a decrease in glutamyl-arginine dipeptide. These fluctuations may be indicative of an imbalance in protein metabolism. A review has indicated that, in osteoarthritis joint tissues, glycolysis and tricarboxylic acid cycle (TCA) pathway activity are enhanced to produce ATP, which is necessary for cartilage repair. In the present study, the differential analysis of H vs. OA also demonstrated an enrichment of energy pathways such as the “TCA cycle.” This finding suggests that, in response to injury in osteoarthritis, chondrocytes enhance ATP synthesis. A general tendency towards an increase in inflammation and energy metabolism-related pathways was observed in the metabolic profile during the transition from H to OA.

**FIGURE 8:**
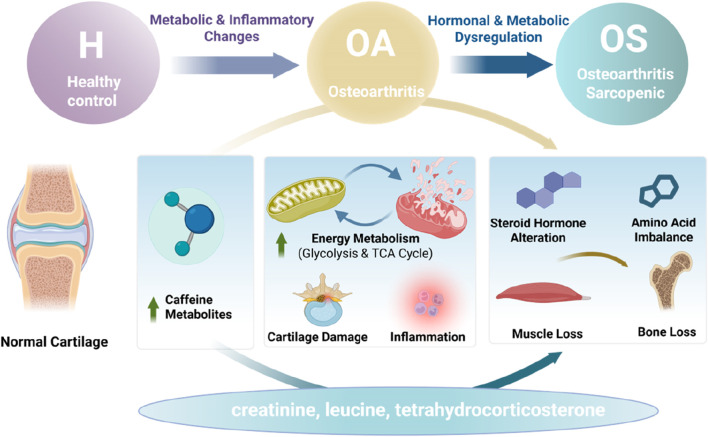
A simplified schematic diagram of the mechanism. Created with BioRender.com.

Progression from OA group to OS group resulted in novel metabolic alterations indicative of a distinct phenotype. Our findings indicate that the steroid hormone biosynthesis pathway and tyrosine metabolism pathway exhibited significant enrichment during the transition from OA group to OS group. At the metabolite level, differential alterations in peptide and lipid metabolites were identified between the OA group and the OS group. Prolyl-histidine dipeptide and 7H-benzo [de]anthrone levels were elevated in the OS group, while phosphatidylinositol [18:0/20:4], indole-3-pyruvate, and leukotriene B3 levels decreased. The observed enrichment of steroid hormone synthesis pathways may be indicative of alterations in sex hormone levels *in vivo* within the OS group. The age-related decline of steroid hormones has been well-established as closely associated with reduced muscle protein synthesis and accelerated catabolism (Huang and Wang, 2021). For instance, decreased hormone levels can accelerate muscle atrophy by inhibiting protein synthesis and promoting protein degradation through signaling pathways such as IGF-1/Akt and MAPK (Sun et al., 2021). Conversely, the enrichment of the tyrosine metabolic pathway suggests a potential involvement of aromatic amino acid metabolism, melanin synthesis, and other related metabolic processes in disease progression. Tyrosine, being the precursor of dopamine and norepinephrine, may exhibit a regulatory impact on neuromuscular function and oxidative stress response through its metabolic dysregulation. These metabolic shifts suggest that sarcopenic osteoarthritis is not merely a more severe stage of osteoarthritis but involves a distinct pathophysiological state characterized by endocrine dysregulation and altered amino acid metabolism. In summary, the metabolic alterations that occur during the transition from the OA group to the OS group appear to be indicative of a distinct metabolic phenotype characterized by endocrine dysregulation, as evidenced by changes in sex hormones and abnormalities in energy and amino acid metabolism. A simplified schematic diagram of the mechanism is provided in Figure 8.

At the biomarker level, the panel of creatinine, leucine, and THB represents a promising diagnostic combination that warrants further investigation in independent cohorts to assess its translational potential. The marked reduction in creatinine levels directly reflects the severe depletion of overall muscle mass in the OS group, serving as a terminal indicator of impaired muscle metabolic function. Creatinine is frequently utilized to evaluate renal function. Its quantification is well-established and consistent, and it is a conventional clinical examination (Ávila et al., 2025). While serum creatinine alone lacks sufficient specificity for diagnosing sarcopenia, derived indices incorporating it demonstrate significant clinical utility. The Sarcopenia Index (serum creatinine/cystatin C ratio) is recommended by the ESCEO expert consensus as a mandatory baseline risk stratification tool in clinical trials (Ladang et al., 2023). A meta-analysis further confirmed that this index provides moderate diagnostic accuracy for sarcopenia (pooled AUC = 0.78), establishing it as a practical and readily accessible screening biomarker (Lin et al., 2024). Metabolomic research directly identified significantly lower plasma creatinine levels in the OS group compared to the H group (Hsu et al., 2024). This finding directly reflects the reduced total muscle mass and impaired muscle metabolic function associated with sarcopenia, leading to its identification as one of twelve potential biomarkers. The association of this metabolite with the OS phenotype suggests a severe anabolic deficit in the OS group, which may be a key endocrine mechanism related to accelerated muscle mass loss.

More critically, the decrease in leucine, the most crucial regulator for activating the mTOR signaling pathway and stimulating muscle protein synthesis, directly points to the core pathology of sarcopenia: anabolic resistance. Based on the synthesis of three key studies, a robust chain of evidence supports leucine’s role as a biomarker for sarcopenia. In a cross-sectional study, it was directly established that lower serum leucine levels are significantly associated with reduced muscle mass and lower grip strength in individuals with type 2 diabetes, confirming its value as a diagnostic biomarker (Dreyer et al., 2008). Through a randomized controlled trial, interventional validation from the reverse angle: leucine supplementation effectively increased appendicular skeletal muscle mass and improved physical function in older adults with sarcopenia, reinforcing the causal link between low leucine levels and muscle loss (Rondanelli et al., 2022).In a large cohort study, they elevated this association to a prognostic level, identifying low leucine levels as a key component of a “metabolic frailty index” that strongly predicts long-term mortality risk in both cardiovascular patients and the general population. In conclusion, serum leucine level not only serves to identify sarcopenia risk and is a viable therapeutic target, but also stands as a significant biomarker for assessing overall metabolic health and predicting long-term survival outcomes (Otvos et al., 2023). This implies that OS not only suffer from reduced muscle mass but also exhibit a severely compromised ability of muscle tissue to respond to anabolic signals.

Furthermore, alterations in serum THB levels in the OS group are highly significant. A marked decrease in THB levels indicates multiple pathophysiological dysregulations within the bodies of OS (McInnes et al., 2004). First, as a key glucocorticoid metabolite with well-established anti-inflammatory effects, reduced THB levels may weaken the body’s endogenous regulation of chronic low-grade inflammation, further exacerbating synovial inflammation and muscle protein breakdown (Moudgil and Venkatesha, 2022). Second, reduced THB levels may reflect abnormalities in glucocorticoid metabolism pathways, leading to excessive accumulation of active cortisol in tissues. This enhances muscle breakdown while inhibiting synthesis, thereby promoting muscle atrophy and joint structural damage (Coury et al., 2019). Existing research indicates that excessive activation of glucocorticoid signaling in cartilage and bone tissues can induce chondrocyte senescence, extracellular matrix degradation, and abnormal bone remodeling (Macfarlane et al., 2023). Although no studies directly link THB to OA or sarcopenia, its association with metabolic syndrome components (Permpoon et al., 2025) and our findings suggest a regulatory role in the inflammation-metabolism-endocrine network underlying musculoskeletal degeneration. Consequently, THB not only represents a potential OS biomarker but also offers new insights into the complex inflammation-metabolism-endocrine network underlying this disease.

The study indicated that creatinine, leucine, and THB possess diagnostic value in distinguishing disease states, with their respective AUC being 0.74, 0.68, and 0.64. Although the discriminatory efficacy of individual biomarkers did not reach the “excellent” level, this aligns with the study’s original intent to identify biomarkers with well-defined biological mechanisms and clinical translational feasibility. These findings provide crucial metabolomic evidence for the early identification and subtype differentiation of OA. The selected markers respectively point to three core pathophysiological dimensions: total muscle mass (creatinine), protein anabolism (leucine), and systemic inflammation (THB). Subsequent work may explore constructing diagnostic models integrating multidimensional indicators to further enhance classification efficacy.

One key finding of this study is that the combined use of creatinine and THB demonstrates significantly greater diagnostic efficacy than either biomarker alone in differentiating between OA and OS. This finding carries substantial biological and clinical implications: creatinine primarily reflects skeletal muscle mass, leucine is closely linked to protein synthesis metabolism, and THB may reflect alterations in the hypothalamic-pituitary-adrenal axis or local inflammatory activity. Together, these three biomarkers characterize the metabolic phenotype of osteosarcopenia across complementary biological dimensions—structural integrity, metabolic activity, and regulatory function. As a result, their combination yields a more comprehensive and robust metabolic profile, which likely accounts for the markedly improved discriminatory power. The clinical utility of this panel is evident in three aspects. First, within the large population of elderly patients with osteoarthritis, this biomarker combination enables objective identification of individuals at preclinical or early stages of sarcopenia. By detecting metabolic dysregulation at an early pathological stage, it overcomes the limitations of conventional assessment methods—which often lack sensitivity to subtle physiological changes—and facilitates timely, prevention-oriented interventions. Second, these results support the potential classification of “metabolic disorder-associated osteoarthritis” as a distinct pathological subtype. Characterized by specific dysregulation of steroid hormones and amino acids, this subtype may exhibit differential responsiveness to targeted nutritional or metabolic therapies, thereby enabling personalized management strategies. Third, the model serves as a reliable adjunct to current diagnostic criteria. When integrated with patient-reported outcomes and physical examinations, it contributes to a multidimensional evaluation framework that enhances both the sensitivity and specificity of osteoarthritis screening.

Its clinical utility is threefold. First, in the vast elderly OA population, this biomarker panel could objectively identify individuals in preclinical or early-stage OS. By detecting metabolic derangements at a nascent stage, it surpasses traditional assessments that often lack sensitivity to early physiological shifts, enabling “prevention-first” precision interventions. Second, our findings support the classification of “metabolic dysregulation-associated OS” as a distinct endotype. This subtype, characterized by specific steroid hormone and amino acid disturbances, may uniquely respond to targeted nutritional or metabolic therapies, facilitating personalized management. Finally, this model serves as a potent adjunct to existing criteria. Integrating it with questionnaires and physical assessments establishes a multidimensional framework, enhancing the sensitivity and specificity of OS screening.

Several limitations persist. The cross-sectional design precludes determining whether these metabolic alterations drive or reflect OS. Additionally, while OS diagnosis followed EWGSOP2 consensus, muscle mass was assessed via BIA rather than the gold-standard DXA, potentially introducing measurement bias. Although we adjusted for BMI and eGFR—strengthening the specificity of our findings—residual confounding from factors like lifestyle and diet cannot be entirely ruled out. Furthermore, the relative homogeneity and modest sample size may constrain generalizability, though these concerns were partially mitigated by rigorous FDR correction and robust validation.

In conclusion, this hypothesis-generating study provides a foundation for objective OS identification. Its findings must be validated in prospective, large-scale, multicenter cohorts. Future research should focus on employing gold-standard diagnostics and conducting longitudinal follow-ups to evaluate these markers’ predictive power for the trajectory of muscle loss and functional decline. Ultimately, establishing standardized cut-offs across diverse populations will be essential for the clinical translation of these OS-specific metabolic signatures.

## 5 Conclusion

In summary, patients diagnosed with OS manifest distinctive metabolic profiles that differentiate them from those with general osteoarthritis. These profiles are characterized by inefficient energy metabolism, enhanced catabolism of multiple pathways, including purines and amino acids, and an accumulation of lipids and inflammation-related metabolites. These changes not only deepen our understanding of the mechanism of interaction between osteoarthritis and sarcopenia, but also provide new ideas and opportunities for diagnostic typing and comprehensive intervention of the disease. The advent of high-level metabolomics studies has led to the anticipation of the identification of more reliable metabolic biomarkers for the early detection and process monitoring of sarcopenia, osteoarthritis, and the development of intervention strategies targeting metabolic imbalances. These developments are expected to improve the functional prognosis and quality of life of patients.

## Data Availability

The raw data supporting the conclusions of this article will be made available by the authors, without undue reservation.
