# Supplementary material for: Creatinine, leucine, and tetrahydrocorticosterone emerge as potential biomarkers for the diagnosis of sarcopenic osteoarthritis in middle-aged and elderly individuals: a cross-sectional exploratory study
Source: Front Mol Biosci. 2026 Feb 11;13:1743095. doi: 10.3389/fmolb.2026.1743095 (PMC12932234; doi:10.3389/fmolb.2026.1743095)
Supplement: Supplementary file 1 [file Supplementaryfile1.docx]

**Supplementary Table S1. Comparison of baseline characteristics between OS participants who provided blood samples (n=22) and those who declined blood sampling (n=8).**

| Characteristic | OS Participants Included (n=22) | OS Participants Declined (n=8) | P-value |
| --- | --- | --- | --- |
| Age, years | 69.2 ± 9.5 | 68.1 ± 8.7 | 0.78 |
| Sex, male (%) | 8 (36.4) | 3 (37.5) | 0.96 |
| BMI, kg/m² | 22.7 ± 3.4 | 23.1 ± 3.1 | 0.75 |
| Kellgren-Lawrence Grade, n (%) |  |  | 0.82 |
| Grade 2 | 10 (45.5) | 4 (50.0) |  |
| Grade 3 | 9 (40.9) | 3 (37.5) |  |
| Grade 4 | 3 (13.6) | 1 (12.5) |  |
| eGFR, mL/min/1.73 m² | 85.6 ± 15.1 | 87.2 ± 13.8 | 0.80 |

**Notes:** Data presented as mean ± standard deviation or number (percentage). BMI: Body Mass Index; eGFR: estimated glomerular filtration rate (CKD-EPI 2009). P-values were derived from an independent samples t-test (Age, BMI, eGFR) or a Chi-square test (Sex, KL Grade). No significant differences were observed (all P > 0.05), indicating no major selection bias on these measured parameters.

**Supplementary Table S2. List of significant differential metabolites after False Discovery Rate (FDR) adjustment (q < 0.05).**

| Metabolite | Comparison | Fold Change (OA/OS or H/OA)* | Regulation | P-value | q-value (FDR) |
| --- | --- | --- | --- | --- | --- |
| Creatinine | OA vs OS | 2.15 | Down | 1.2E-04 | 0.012 |
| Leucine | OA vs OS | 1.92 | Down | 3.5E-04 | 0.028 |
| Tetrahydrocorticosterone | OA vs OS | 0.48 | Up | 7.8E-04 | 0.041 |
| Theobromine | H vs OA | 0.32 | Up | 4.1E-06 | 0.001 |
| N-Methylnicotinamide | H vs OA | 2.85 | Down | 2.3E-05 | 0.003 |
| Glutamylarginine | H vs OA | 2.10 | Down | 1.1E-04 | 0.010 |

**Notes:** This table presents a subset of metabolites that remained significant after Benjamini-Hochberg FDR adjustment (q < 0.05). The three key candidate biomarkers for sarcopenic osteoarthritis are shown.
*Fold Change (FC) is expressed as the ratio of the mean intensity in the first-named group relative to the second (e.g., for OA vs OS, FC = Mean(OA)/Mean(OS); FC > 1 indicates down-regulation in OS, FC < 1 indicates up-regulation in OS).

**Supplementary Table S3. Following adjustment for BMI and eGFR as covariates, the differences in serum creatinine, leucine, and total haemoglobin levels between the OA group and the OS group remained statistically significant (q < 0.05).**

| Metabolite | Comparison | Covariate | 1. value   (Before adjustment) | P-value(Adjusted) |
| --- | --- | --- | --- | --- |
| Creatinine | OA vs OS | BMI | 0.014 | 0.002 |
| Leucine | OA vs OS | BMI | 0.026 | 0.014 |
| Tetrahydrocorticosterone | OA vs OS | BMI | 0.049 | 0.029 |
| Creatinine | OA vs OS | eGFR | 0.014 | 0.008 |

**Notes:** BMI: Body Mass Index; eGFR: estimated glomerular filtration rate (CKD-EPI 2009). Following covariate adjustment for BMI, the differential levels of creatinine, leucine, and THB between the OA and OS groups remained statistically significant. Following adjustment for covariates in eGFR, the differential levels of creatinine between the OA and OS groups remained statistically significant.
